# Outcomes and success predictors of micropulse transscleral cyclophotocoagulation in patients with refractory glaucoma

**DOI:** 10.5935/0004-2749.2024-0340

**Published:** 2025-09-10

**Authors:** Fábio Nishimura Kanadani, Júlia Maggi Vieira, Larissa Fouad Ibrahim, Senice Alvarenga Rodrigues Silva, Syril Dorairaj, Tiago Santos Prata

**Affiliations:** 1 Glaucoma Institute, Belo Horizonte, MG, Brazil; 2 Mayo Clinic, Jacksonville, Florida, USA; 3 Instituto de Olhos Ciências Médicas, Belo Horizonte, MG, Brazil; 4 Department of Ophthalmology, Mayo Clinic, Jacksonville, Florida, USA; 5 Universidade Federal de São Paulo, São Paulo, SP, Brazil

**Keywords:** Intraocular pressure/physiology, Glaucoma, open-an gle/surgery, Trabeculectomy, Laser coagulation/methods, To nometry, ocular/methods, Postoperative complications, Anti hypertensive agents/therapeutic use

## Abstract

**Purpose:**

This study aimed to report the surgical outcomes and success predictors of micropulse transscleral cyclophotocoagulation in eyes with refractory glaucoma.

**Methods:**

This was a noncomparative, interventional case series. Patients with refractory glaucomas, defined as eyes with prior incisional glaucoma surgery failure and uncontrolled intraocular pressure, who underwent micropulse transscleral cyclophotocoagulation between March 2017 and June 2021 were enrolled. A minimum follow-up period of 6 months was required. Preoperative and postoperative intraocular pressure, number of hypotensive medications, surgical complications, and any subsequent related events were recorded. Success criteria were as follows: 1) intraocular pressure reduction ≥20% and intraocular pressure ≤18 mmHg; 2) intraocular pressure reduction ≥30% and intraocular pressure ≤15 mmHg. The need for topical hypotensive medications was not considered a failure.

**Results:**

Seventy-nine (79) eyes (79 patients; mean age, 57.5 ± 20.6 years) were included. Overall, the median follow-up duration was 12.0 (interquartile interval, 6–24) months, and the mean intraocular pressure was reduced from 22.8 ± 6.8 mmHg to 15.5 ± 5.6 mmHg at the last follow-up visit (p<0.001). The mean number of medications was reduced from 2.8 ± 0.7 to 2.0 ± 1.0 (p<0.01). At 12 months postoperatively, the success rates for criteria 1 and 2 were 54.9% and 49.7%, respectively. Aside from one case of corneal ulcer, which fully resolved with clinical treatment, and two cases of persistent hypotony (with no visual acuity loss during follow-up), no other vision-threatening complications were observed during the postoperative period. The magnitude of intraocular pressure reduction at 1 month (adjusted to preoperative intraocular pressure; HR=1.01; p=0.002).

**Conclusion:**

Our findings suggest that micropulse transscleral cyclophotocoagulation is a relatively effective alternative for managing refractory glaucomas, with minor postoperative complications. In addition, the initial intraocular pressure reduction was a statistically significant predictor of 1-year success in patients undergoing micropulse transscleral cyclophotocoagulation.

## INTRODUCTION

Glaucoma is the leading cause of irreversible blindness worldwide^(1^,^2)^. It is a progressive neuropathy with an uncertain pathophysiology but known risk factors, such as elevated intraocular pressure (IOP)^(3^,^4)^. Currently, the primary treatment consists of controlling IOP with hypotensive medications, lasers, conventional filtering surgeries, and minimally invasive procedures (MIGS)^(1^,^5)^.

Although most patients achieve glaucoma control with laser trabeculoplasty or topical hypotensive medications, some require surgical interventions. In patients with early or moderate glaucoma, MIGS may be a reasonable surgical option^(1^,^6^,^7)^. In. Other surgical pro cedures should be considered in eyes with progressive and/or advanced glaucoma. For decades, trabeculectomy (TRAB) has been the primary surgical treatment for uncontrolled glaucoma^(8)^. This procedure aims to reduce IOP by increasing aqueous humor (AH) outflow into the sub-Tenon space. However, some eyes are refractory to TRAB and may need further intervention. In this context, other techniques that reduce IOP by decreasing AH production have been described^(9^,^10)^.

Transscleral cyclophotocoagulation (TSCPC) uses a continuous diode laser to target the pigmented ciliary body epithelium and stroma, reducing AH produc tion^(11)^. However, it may lead to serious complications, such as chronic hypotony, prolonged inflammation of surrounding tissues, phthisis, and others^(1^,^6^,^9^,^12)^. Micropulse TSCPC (MP-TSCPC) has been proposed as a less aggressive alternative to minimize these side effects. This technique uses repetitive, short “on” and “off” diode laser cycles, allowing effective treatment of the ciliary body with minimal damage to adjacent tissues^(1^,^6^,^9^,^13)^. Therefore, MP-TSCPC may be considered an alternative to drainage implant surgeries in refractory glaucoma cases, which are more surgically challenging and require closer follow-up^(14)^. In this study, we aimed to investigate the efficacy and safety of MP-TSCPC in eyes with refractory glaucoma and to identify possible predictors of success.

## METHODS

This was a noncomparative, single-center, interventional case series. The study followed the principles of the Declaration of Helsinki and was approved by the ethics committees of *Instituto de Olhos Ciências Médicas* and *Universidade Federal de São Paulo* (UNIFESP).

### Patients

We reviewed the records of all patients with refractory glaucomas–defined as eyes with prior incisional glaucoma surgery failure and uncontrolled IOP–who underwent MP-TSCPC between March 2017 and June 2021 at the Medical Science Eye Institute, Belo Horizonte, Brazil. All procedures were performed by a single surgeon (FK). Only patients with at least 6 months of follow-up were included. If both eyes were eligible, the right eye was arbitrarily selected for analysis. Data collected included preoperative and postoperative IOP, number of antiglaucoma medications, surgical complications, visual field results, and any related events or additional procedures.

### Success criteria

Two criteria were considered: 1) IOP reduction ≥20% and IOP ≤18mmHg; 2) IOP reduction ≥30% and IOP ≤15 mmHg. The use of topical hypotensive medications was not considered a failure. Failure was defined as not meeting either criterion at two consecutive visits, with at least 3 months of follow-up. Hypotony (IOP <6 mmHg) was also considered a failure, and such eyes were excluded from the final mean IOP calculation. Any need for additional surgical intervention at any time was also classified as failure.

### Description of the laser technique

Before the procedure, patients received 2% lidocaine gel and a caruncular injection of 3 mL of 0.75% bupivacaine and 2% xylocaine. All patients were also given sedation and IV analgesia with propofol and dipyrone. An 810-nm laser (Iridex Corporation, Mountain View, CA, USA) with the MicroPulse probe was used, applied with 2% methylcellulose. Treatment consisted of 90 seconds superiorly and 90 seconds inferiorly (swing time: 10 seconds), followed by two additional 45-second cycles in each region. The laser was set to 2000 mW with a 31.3% duty cycle. The treated area included the upper and lower 180° of the eye, excluding the 3 and 9 o’clock positions.

### Postoperative

During the postoperative period, 1% prednisolone acetate was prescribed four times daily with gradual tapering based on follow-up evaluations, along with 1% atropine twice daily for 10 days. On the first postoperative day, oral acetazolamide was discontinued based on the measured IOP. At each visit, glaucoma medications were continued or withdrawn at the physician’s discretion, depending on IOP levels and glaucoma severity.

### Statistical analysis

Descriptive analysis was used to present demographic and clinical data. The D’Agostino-Pearson test was applied to assess normality. Normally distributed data were expressed as mean (± standard deviation), and nonnormally distributed data as median (interquartile interval). Continuous variables were compared using the paired t-test or Wilcoxon signed-rank test, according to distribution. Kaplan–Meier survival analysis estimated success rates at defined postoperative timepoints. Survival probability at 12 months was compared among glaucoma subtypes using the log-rank test. Cox proportional hazards regression analyzed the effect of risk factors on survival, including age, glaucoma severity (visual field mean deviation; VFMD), glaucoma type, number of prior surgeries, number of glaucoma medications, and magnitude of IOP reduction at one month, adjusted for baseline IOP. Statistical analysis was performed using MedCalc software (MedCalc Inc., Mariakerke, Belgium), with significance set at p<0.05.

## RESULTS

Seventy-nine eyes from 79 patients (mean age, 57.5 ± 20.6 years) were included. The most common type of glaucoma was primary open-angle (n=51, 64.6%), followed by congenital (n=18, 22.8%) and secondary glaucoma (n=10, 12.6%). The median number of previous surgeries was 1.6 (interquartile interval, 1.2–2.0). The mean visual field (mean deviation) was 20.86 ± 8.31 dB. Table 1 provides detailed baseline characteristics.

**Table 1:** Patients’ demographic and clinical characteristics

Parameters	Group (n=79)
Age (years) *	57.5 ± 20.6
Sex (male/female)	39/40
POAG	51 (64.6%)
Secondary glaucoma	10 (12.6%)
Congenital glaucoma	18 (22.8%)
Baseline IOP (mmHg) *	22.8 ± 6.8
Baseline medications *	2.8 ± 0.7
Baseline Acetazolamide	34 (43%)
Follow-up (months) **	12.0 (6-24)
Previous glaucoma surgeries **	1.6 (1.2 - 2.0)

* Data with normal distribution are presented as mean ± SD whenever indicated.

** Data with non-normal distribution are presented as median (interquartile interval).

POAG= primary open-angle glaucoma; IOP= intraocular pressure.

All patients were receiving topical ocular hypotensive medications, and 34 (43.0%) were also receiving oral acetazolamide. All eyes had undergone previous incisional glaucoma surgery, with 29.1% having two prior procedures and 21.5% having three or more.

**Figure 1:**
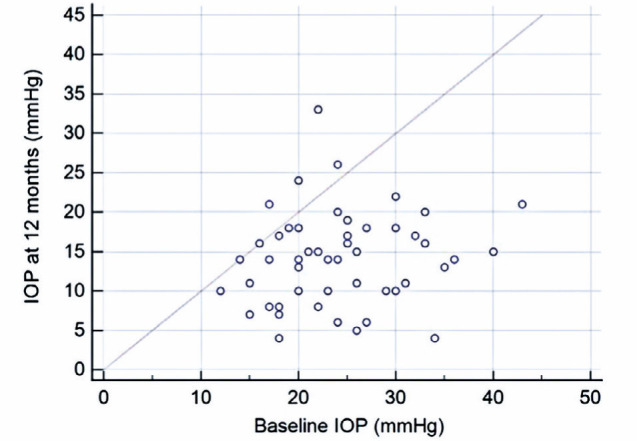
Baseline IOP x IOP at 12 months. IOP= intraocular pressure.

The median follow-up duration was 12.0 months (interquartile interval, 6–24). The mean IOP significantly decreased from 22.8 ± 6.8 mmHg (range, 11–43) to 15.5 ± 5.6 mmHg (range, 3–30) at the last follow-up visit (p<0.001), representing a 32% IOP reduction (Figure 1). The mean number of hypotensive eyedrops decreased from 2.8 ± 0.7 preoperatively to 2.0 ± 1.0 at the last follow-up visit (p<0.001).

**Figure 2:**
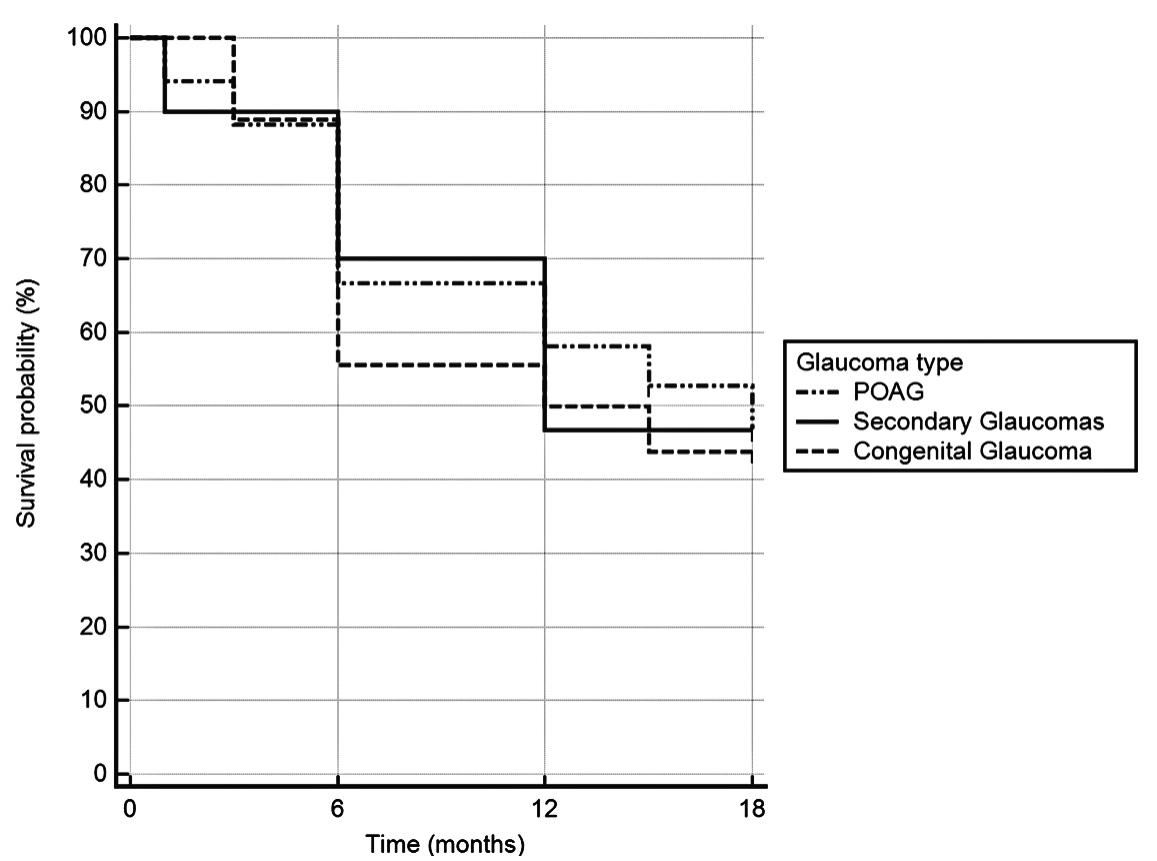
One-year success rates by glaucoma type showed no significant differences in survival probability at 12 months, as determined by the log-rank test.

The success rates for criterion 1 were 64.6% at 6 months, 54.9% at 12 months, and 46.6% at 18 months. For criterion 2, success rates were 60.8% at 6 months, 49.7% at 12 months, and 34.5% at 18 months. The number of eyes analyzed at each timepoint were: 79 at 6 months, 58 at 12 months, and 41 at 18 months. At 12 months, success rates for criterion 1 by glaucoma type were 58.1% in the primary open-angle glaucoma (POAG) group, 46.7% in the secondary glaucoma group, and 50.0% in the congenital glaucoma group (Figure 2). The log-rank test showed no statistically significant differences in survival curves between groups (p>0.05).

The magnitude (%) of IOP reduction in the first month was significantly correlated with success rates (adjusting for baseline IOP; hazard ratio 1.01; p=0.0021). Each additional 10% initial IOP reduction improved the success rates by 10%. Age, VFMD, number of previous surgeries, type of glaucoma, and number of medications were not significant in the model (p≥0.26). In addition to one case of corneal ulcer, which fully recovered with clinical treat ment, and two cases of persistent hypotony (with no visual acuity loss during follow-up), no other vision-threatening complications were observed postoperatively.

## DISCUSSION

The management of refractory glaucoma is challenging and often requires multiple surgical procedures. In daily practice, we often manage eyes with poor vision and poor ocular prognosis, not only due to glaucoma severity but also due to incisional procedures^(15)^. In this context, MP-TSCPC emerged as a way to reduce IOP while offering a better safety profile than conventional surgeries. After evaluating nearly 80 eyes with refractory glaucomas, we found an average pressure reduction of 30%, a positive impact on medication use, and minimal significant side effects. These results are clinically relevant, especially considering the severity of the operated eyes, with 35% having secondary or congenital glaucomas and 50% having two or more prior incisional surgeries.

Our study evaluated 80 patients with refractory glau coma who underwent MP-TSCPC, with a median follow-up of 12.0 months. The established success rate–IOP reduction by at least 20% and less than or equal to 18 mmHg (criterion 1)–was achieved in over 50% of patients at 12 months, slightly lower than in studies reporting higher rates. Using the strictest criterion, the success rate was 49.7% at the same time point. Despite including only refractory cases, more than half of the patients responded well at 12 months, regardless of the criteria. Among POAG patients, we achieved a 58.1% success rate. Several MP-TSCPC studies report success rates between 66% and 80%, depending on glaucoma type and severity, baseline IOP, and success criteria^(6^,^9^,^16^-^18)^. Although not directly comparable, our findings support those previously published.

We also aimed to identify predictors of MP-TSCPC success. After adjusting for baseline IOP, only the magnitude of IOP reduction in the first month was significantly associated with outcomes at the last visit (hazard ratio 1.01; p=0.0021). The predictive value of early res ponse is important for managing these cases.

Regarding safety, MP-TSCPC showed an excellent profile, with only one case of corneal ulcer, which fully resolved with medical treatment. Our results align with those of Bendel and Patterson, who reported no complications^(7^,^19)^. However, some studies noted complications such as hypotonia, phthisis bulbi, persistent inflammation, and macular edema^(1^,^17^,^20^-^22^). We believe our low complication rate may be due to the more conservative laser protocol (lower total energy settings) used to enhance safety. Still, other studies reported favorable efficacy-to-safety ratios using higher energy protocols. Magacho et al. conducted a retrospective study involving 84 eyes without prior glaucoma surgery and 101 eyes with a history of surgery. Surgical success rates were 92.9% and 87.1%, respectively, with similar criteria. The first group had one case of cystoid macular edema and 13 eyes with persistent mydriasis. The second group had one case of hypotonia and two of phthisis bulbi, both associated with neovascular glaucoma^(23)^. Another study by the same group showed the effectiveness and safety of Double-Session MP-TSCPC in 89 eyes, with no hypotonia. That study reported two eyes with persistent mydriasis and three with decreased visual acuity due to cystoid macular edema, corneal edema, and one case of glaucoma worsening^(24)^. Both studies supported Double-Session MP3 as a safe, effective option for treatment-naïve and refractory eyes^(23^,^24)^.

Our study has specific characteristics and limitations. First, it was a retrospective, single-center study. Second, our sample size may have limited comparisons among glaucoma subtypes. Third, longer follow-up is needed to assess long-term efficacy and side effects. Lastly, all patients received the same laser protocol; an individua lized approach may have produced better outcomes.

In conclusion, MP-TSCPC appears to be a relatively effective treatment for glaucomas, with minimal postoperative complications. Additionally, early IOP reduction was a good predictor of 1-year success. Further prospective studies with individualized protocols and longer follow-up are warranted to better determine MP-TSCPC’s role, not only in refractory glaucomas but also in eyes with good visual prognosis.

## Data Availability

The data correspond to patient records; thereforer, it cannot be publicly exposed to preserve the confidentiality of those involved.
